# Transcriptome and 16S rRNA Analyses Reveal That Hypoxic Stress Affects the Antioxidant Capacity of Largemouth Bass (*Micropterus salmoides*), Resulting in Intestinal Tissue Damage and Structural Changes in Microflora

**DOI:** 10.3390/antiox12010001

**Published:** 2022-12-20

**Authors:** Zhuo Song, Wei Ye, Yifan Tao, Tao Zheng, Jun Qiang, Yan Li, Wenting Liu, Pao Xu

**Affiliations:** 1 Wuxi Fisheries College, Nanjing Agricultural University, Wuxi 214081, China; 2 Key Laboratory of Freshwater Fisheries and Germplasm Resources Utilization, Ministry of Agriculture and Rural Affairs, Freshwater Fisheries Research Center, Chinese Academy of Fishery Sciences, Wuxi 214081, China

**Keywords:** dissolved oxygen, *Micropterus salmoides*, transcriptome, 16S rDNA, intestine

## Abstract

Dissolved oxygen (DO) is a key factor affecting the health of aquatic organisms in an intensive aquaculture environment. In this study, largemouth bass (*Micropterus salmoides*) were subjected to acute hypoxic stress for 96 h (DO: 1.00 mg/L) followed by recovery under sufficient DO conditions (DO: 7.50 mg/L) for 96 h. Serum biochemical indices, intestinal histomorphology, the transcriptome, and intestinal microbiota were compared between hypoxia-treated fish and those in a control group. The results showed that hypoxia caused oxidative stress, exfoliation of the intestinal villus epithelium and villus rupture, and increased cell apoptosis. Transcriptome analyses revealed that antioxidant-, inflammation-, and apoptosis-related pathways were activated, and that the MAPK signaling pathway played an important role under hypoxic stress. In addition, 16S rRNA sequencing analyses revealed that hypoxic stress significantly decreased bacterial richness and identified the dominant phyla (Proteobacteria, Firmicutes) and genera (*Mycoplasma*, unclassified *Enterobacterales*, *Cetobacterium*) involved in the intestinal inflammatory response of largemouth bass. Pearson’s correlation analyses showed that differentially expressed genes in the MAPK signaling pathway were significantly correlated with some microflora. The results of this study will help to develop strategies to reduce damage caused by hypoxic stress in aquacultured fish.

## 1. Introduction

Dissolved oxygen (DO) is an important environmental factor affecting the growth and development of aquatic organisms. In the process of fish farming, especially in overcast and rainy weather in summer and autumn, acute hypoxia can readily occur. Prolonged low DO levels (below 1–2 mg/L) adversely affect fish [1]. Low DO not only affects fish behavior, immunity, and metabolism [2] but also leads to oxidative stress [3] and even death [4]. Thus, hypoxia seriously restricts the sustainable development of intensive aquaculture.

Previous studies have revealed the effects of the duration of hypoxia on oxidative stress, inflammation, and apoptosis, in aquatic organisms. For example, culturing large yellow croaker (*Larimichthys crocea*) in water with a DO level of 2 mg/L for 96 h can cause oxidative stress, affect the balance of the redox system, and lead to gill tissue damage [5]. A study on hybrid yellow catfish (*Pelteobagrus fulvidraco* ♀ × *Pelteobagrus vachelli* ♂) found that oxidative stress, respiratory metabolism, and apoptosis were affected when the DO level reached 0.7 ± 0.05 mg/L and was maintained at that level for 6.5 h [6]. The contents of serum metabolites (glucose and lactic acid) in mrigal (*Cirrhinus mrigala*) also changed significantly upon 24 h of exposure to water with a DO level of 0.5 ± 0.04 mg/L [7]. In common carp (*Cyprinus carpio*), hypoxia led to an inflammation response, and then the reintroduction of oxygen into anoxic tissue caused oxidative stress and immune damage to fish tissue [8].

The intestine is not only an important part of the digestive and absorption system of fish but also an important immune organ. Under hypoxic stress, the intestinal mucosal immune system is activated and drives the adaptation to hypoxia by upregulating the expression of several metabolic enzymes and vasoactive factors and disrupting barrier function, growth, and apoptosis [9,10]. The disruption of the oxygen gradient is responsible for many intestinal diseases [11]. Several studies have shown that hypoxic stress significantly affects the intestinal structure of fish. Dong et al. found that intermittent hypoxia for 7 days led to the exfoliation of intestinal epithelial cells and a significant decrease in the length of intestinal villi [12]; Yang et al. found that the height and width of intestinal villi and the thickness of the muscle layer decreased significantly after 28 days of hypoxic stress [13]. Hypoxia also affects the immune system of fish, and was shown to affect neutrophils and cause inflammation in the intestinal mucosa of *Salmo salar* [9] and increase oxidative stress and apoptosis-related factors in the intestine of *Lateolabrax maculatus* [14]. Therefore, exposure to hypoxia can severely affect the intestinal health of fish.

The rapid development of second-generation sequencing technologies and bioinformatic analysis methods has allowed for effective and rapid analyses of the molecular mechanisms underlying responses of various tissues and organs to environmental conditions [15]. Using these techniques, researchers have unraveled a series of molecular changes in fish under hypoxic stress. It has been reported that in channel catfish (*Ictalurus punctatus*) [16] and blunt snout bream (*Megalobrama amblycephala*) [17], the pathways significantly enriched with differentially expressed genes (DEGs) under hypoxic stress are HIF-1, MAPK, and PI3K-Akt. The environmental stress caused by hypoxia reduces the oxygen transfer to the surrounding tissues. Previous analyses have shown that hypoxic stress also reduces the abundance of beneficial bacteria and increases the abundance of opportunistic pathogens in *Pelteobagrus vachelli* [18], thus leading to a higher incidence of gastrointestinal and intestinal inflammation. Studies on oriental river prawn (*Macrobrachium nipponense*) showed that intestinal microorganisms had important effects on physiological homeostasis under hypoxia [19]. Intestinal microbes and hosts can interact with each other [20,21]. We have confirmed this in hybrid yellow catfish (*Tachysurus fulvidraco* ♀ *× Pseudobagrus vachellii* ♂) [22]. To determine whether a similar phenomenon would occur under hypoxic conditions, it is necessary to study the correlation between the gut microbiota and the transcriptome. The Largemouth bass (*Micropterus salmoides*) is popular because of its delicious meat, lack of intermuscular spines, and high nutritional value. In the study of hypoxia, 6 and 96 h are common stress times [23,24]. To observe the dynamic physiology of largemouth bass under hypoxic stress, we set several time points in multiples of four to determine the characteristics of stress response in largemouth bass. We identified changes in intestinal molecular indices by transcriptome sequencing and analyzed the intestinal histomorphology, serum antioxidant capacity, and intestinal microbial composition under acute hypoxic stress followed by reoxygenation. These results contribute to a comprehensive understanding of how these fish adapt to hypoxia, and will be useful to devise strategies to reduce the damage caused by hypoxic stress in cultured fish.

## 2. Materials and Methods

### 2.1. Fish Maintenance

The fish used in the experiment were healthy largemouth bass that were free of disease and injuries from the Yixing Base of Freshwater Fisheries Center, Chinese Academy of Fishery Sciences (Wuxi, China). Before the experiment, the fish were temporarily reared for 15 days in a 400 L indoor tank (water temperature 27 °C ± 1 °C, DO > 7.5 mg/L, ammonia nitrogen and nitrite < 0.01 mg/L, pH 7.6 ± 0.2). They were fed a commercial diet at 3% of their bodyweight (46% crude protein, 6% crude fat) at 8:00 and 16:00, respectively.

### 2.2. Determination of 96h-LH50

According to our previous research [25], fish (average weight, 8.27 ± 1.20 g) were subjected to DO at five different levels (0.15, 0.3, 0.6, 1.2, 2.4 mg/L) for 96 h to determine the median lethal hypoxia (96h-LH_50_). The preliminary experiment was carried out in a 400 L indoor tank (20 fish per tank, with a total of 300 fish). Each inflatable tank was filled with 150 L tap water, and the oxygen concentration was measured with a DO meter (LD0101 probe, range: 0.1–20.0 mg/L, Hach, Loveland, LVLD, USA). Before the start of the experiment, the DO was adjusted to the specified level using nitrogen. The fish were observed every half hour. At each observation time, the dead fish were counted and removed. The mortality of largemouth bass was recorded at 24 h, 48 h, 72 h, and 96 h, and the cumulative mortality in each treatment during 96 h was calculated. Finally, the 96h-LH_50_ of largemouth bass was obtained by linear interpolation.

### 2.3. Sample Collection

The hypoxic stress experiments were carried out on the basis of 96h-LH50 for 96 h. The control group (DO: 7.50 mg/L) and the hypoxia group (DO: 1.00 mg/L) were set up with three parallel experiments per treatment group. The experiment was carried out in six 400 L culture tanks, each one containing 60 uniform and healthy largemouth bass (8.27 ± 0.22 g). In the hypoxia groups, the DO level was maintained at 1.00 mg/L for 96 h by adding nitrogen along with oxygen through the inlet valve. At the end of 96 h, oxygen was supplied through the valve to quickly return the DO to the normal level (DO: 7.50 mg/L), and these conditions were maintained for a further 96 h. During the experiment, the fish were fed normally.

Samples were taken at 0 h, 6 h, 24 h, 96 h under hypoxic stress (DO: 1.00 mg/L) and after 96 h of recovery under sufficient DO conditions (DO: 7.50 mg/L). At each sampling time, 12 fish were randomly selected from each of the three parallel groups in the hypoxia group and the control group for tail vein blood collection and foregut extraction. The blood was collected by caudal vein drawing. As described by Ma et al., [26], the serum was immediately centrifuged (4 °C, 3000× *g*) and frozen at −20 °C. These serum samples were used to analyze biochemical parameters. The foregut samples were fixed in 4% paraformaldehyde solution for pathological observations and apoptotic cell detection. Another 12 fish were taken from each of three parallel groups: 0 h group (Ctrl), 96 h group (Hyp), and 96 h recovery group (Rec). Foregut tissue samples were collected and frozen in liquid nitrogen and stored at −80 °C. The foregut of each fish was divided into three parts, which were used for 16S rRNA sequencing, transcriptome sequencing, and qRT-PCR verification, respectively.

### 2.4. Determination of Serum Biochemical Indices

The serum supernatant was used to analyze the contents of glucose, lactic acid (LAC), and malondialdehyde (MDA) and the activity of superoxide dismutase (SOD) and catalase (CAT). All these analyses were conducted using kits from the Shanghai Langton Biology Co., Ltd. (Shanghai, China) [25]. The absorbance values of the reaction mixtures were determined using an Epoch™ Microplate Spectrophotometer (BioTek Instruments, Inc., Winooski, VT, USA).

### 2.5. Histopathological Analysis of Intestine Samples

Paraffin sections were prepared as described previously [27]. The intestinal samples were fixed in 4% paraformaldehyde solution for 24 h, dehydrated in an ethanol gradient, then cleared in a mixture of xylene:anhydrous ethanol = 1:1 and xylene. The transparent samples were embedded in wax blocks using a Leica EG1150H embedding machine (leica, Germany), and the wax blocks were cut into thin slices (7 μm thickness) using a Leica RM2255 slicer (leica, Germany). After spreading, drying, and baking, the paraffin slices were dewaxed in xylene and ethanol solutions, and then stained with hematoxylin–eosin (HE). Finally, the slices were sealed with neutral gum and observed and photographed under a Nikon Eclipse Ci microscope (Nikon, Tokyo, Japan) equipped with a Nikon digital sight DS-FI2 imaging system. The length and width of villi and the thickness of the muscle layer in each slice were measured using Image-Pro Plus 6.0 software (media cybernetics, Silver Spring, MD, USA).

### 2.6. Detection of Intestinal Cell Apoptosis in Largemouth Bass

Intestinal cell apoptosis was detected using a TUNEL (terminal dUTP nick-end labeling) kit (Yanjin Biotechnology Co., Ltd. Shanghai, China). After the paraffin sections were prepared as described above, they were immersed in xylene I (20 min), xylene II (20 min), and ethanol (100, 100, 85, 75%; 5 min at each concentration), and then washed with water for 2 min. The tissues were covered with DNase-free proteinase K (20 μg/mL), incubated at 37 °C for 25 min, and washed with PBS three times for 5 min each time. Tissue samples were placed in a wet box, covered with TUNEL reaction solution, and incubated at 37 °C for 1 h. After incubating with 3,3-diaminobenzidine (DAB) for 5 min, sections were restained with hematoxylin for 12 s. The sections were observed and photographed under a Nikon Eclipse Ci microscope equipped with a Nikon DS-U3 imaging system. The nuclei of normal nonapoptotic cells were stained blue by hematoxylin, while the nuclei of positive apoptotic cells were stained brownish-green by DAB. Image-Pro Plus 6.0 were used to calculate the number of apoptotic cells.

### 2.7. Sequencing and Analysis of Intestinal Transcriptome

#### 2.7.1. Construction and Sequencing of mRNA Libraries

Total RNA was extracted from intestinal samples of the Ctrl, Hyp, and Rec groups (*n* = 12) using a TRIzol kit (Invitrogen, Carlsbad, CA, USA). The quantity and purity of total RNA were determined using a NanoDrop ND-1000 instrument (NanoDrop, Wilmington, DE, USA) and the integrity of RNA was tested using a Bioanalyzer 2100 instrument (Agilent, Palo Alto, CA, USA). Equal amounts of RNA were extracted from the Ctrl group, Hyp group, and Rec group and mixed for sequencing. There were three replicates in each group, and a total of nine samples were prepared (Ctrl1, Ctrl2, Ctrl3, Hyp1, Hyp2, Hyp3, Rec1, Rec2, Rec3). The mRNA with polyA (polyadenylate) was specifically captured using Dynabeads Oligo magnetic beads (Dynabeads Oligo (dT), cat.25-61005, Thermo Fisher, Waltham, MA, USA) through two rounds of purification, and then RNA-Seq libraries were constructed using the mRNA-Seq sample preparation kit (Illumina, San Diego, CA, USA) [28]. Finally, libraries were sequenced on the Illumina Novaseq sequence 6000 platform (LC Bio Technology Co., Ltd. Hangzhou, China) in the PE150 sequencing mode.

#### 2.7.2. Data Filtering, Read Mapping and Detection of Differentially Expressed Genes

Cutadapt [29] was used to remove sequencing connectors and low-quality sequencing data, and Hisat2 [30] was used to compare the preprocessed effective data (clean data) with the reference genome of the largemouth bass (https://www.ncbi.nlm.nih.gov/genome/?term=Micropterus+salmoides%5Borgn%5D (accessed on 21 October 2021)). According to the alignment results, the transcripts were reconstructed using Stringtie [31] and the expression levels of all genes in each sample were calculated. The FPKM value [32] (fragments per kilobase million) was used as the measurement index of gene expression to calculate gene expression in different samples. T-tests were used to detect significant differences in gene expression between pairs of samples. Differentially expressed genes were screened using the following criteria: | log2FOLDCHANGE | > = 1 & *p* < 0.05. Gene ontology (http://geneontology.org/, accessed on 20 November 2021, GO) and Kyoto Encyclopedia of Genes and Genomes (http://www.kegg.jp/, accessed on 20 November 2021, KEGG) enrichment analyses were used to determine the function and related metabolic pathways of DEGs, respectively.

#### 2.7.3. Identification of DEGs in Intestines by qRT-PCR

Total RNA was extracted from intestines of Ctrl, Hyp, and Rec groups using an RNAiso Plus kit (TaKaRa, Dalian, China). The mass and concentration of RNA were determined by measuring the absorbance values of 260 nm and 280 nm (A260/A280 ratio, 1.9–2.1). A Prime Script template RT Master Mix (Takara) (Dalian, China) kit was used to synthesize the template cDNA by reverse transcription.

All the primers (Table S1) used to amplify differentially expressed genes were synthesized by the Genewiz Biotechnology Co., Ltd. (Suzhou, China). Using the known *β-actin* gene of largemouth bass as the internal reference [33], real-time fluorescence quantitative PCR analyses were performed using the SYBR ^®^Premix Ex Taq assay (Takara, Dalian, China) kit. The composition of the qRT-PCR reaction system was as follows: 2 × SYBR Premix Ex Taq™ 12.5 μL, 1 μL each of the forward and reverse primers, 50 × ROX reference Dye II 0.5 μL, 2 μL cDNA template, and RNase-free dH_2_O to complete the volume to 25 μL. The thermal cycling conditions were as follows: 95 °C for 30 s; 95 °C for 5 s; 60 °C for 30 s (40 cycles). To confirm the specificity of primers, the amplification curve and melting curve of PCR were confirmed at the end of each operation. According to the Ct value measured by fluorescence quantitative PCR, the relative gene transcript level was calculated by the 2^−∆∆Ct^ method [34].

### 2.8. Sequencing and Analysis of Intestinal Microorganisms in 16S rRNA

#### 2.8.1. DNA Extraction and Sequencing

DNA was extracted from the foregut of the Ctrl, Hyp, and Rec groups using an EZNA ^®^ Stool DNA Kit (D4015, Omega Inc., Norcross, GA, USA). Ten samples with good DNA quality were selected from each group for follow-up experiments. The V4 and V3 variable regions of the bacterial 16s rRNA gene (400–450 bp) were selected for PCR amplification. The PCR thermal cycling program was as follows: 98 °C initial denaturation for 30 s, then 98 °C for 10 s (denaturation), 54 °C for 30 s, then 35 cycles of 72 °C for 45 s (annealing), and final extension for 10 min at 72 °C. The PCR products were purified using AMPure XT beads (Beckman Coulter Genomics, Danvers, MA, USA) and quantified by Qubit (Invitrogen, Carlsbad, CA, USA). The PCR amplification products were detected by 2% w/v agarose gel electrophoresis and recovered using AMPure XT beads recovery reagent. The purified PCR products were evaluated using an Agilent 2100 Bioanalyzer (Agilent, CA, USA) and an Illumina (Kapa Biosciences, Woburn, MA, USA) library quantification kit. Qualified library concentrations were higher than 2 nM. After gradient dilution of the qualified sequencing libraries, the libraries were mixed according to the required sequencing quantity and denatured into single strands by NaOH for sequencing. Double-ended sequencing of 2 × 250 bp was carried out using a NovaSeq 6000 sequencer (LC Bio Technology Co., Ltd. Hangzhou, China), with reagents of the NovaSeq 6000 SP Reagent Kit (500 cycles).

#### 2.8.2. Statistical and Bioinformatic Analysis

After sequencing, the raw double-terminal data were spliced by overlapping, and then quality-control steps and chimera filtering were conducted to obtain high-quality clean data. Then, we used the concept of ASVs (amplicon sequence variants) to construct the OTUs (operational taxonomic units) table, and obtained the final ASV feature table and feature sequences for further analyses. QIIME (v.1.8.0) was used to calculate goods coverage, observed species, Chao1, and Shannon’s and Simpson’s indices to evaluate the alpha diversity of samples and to determine differences in species composition among samples. SILVA (release 138, https://www.arb-silva.de/documentation/release-138 (accessed on 1 November 2021)) and the NT-16S database were used to classify species and compare abundance ratios. The results are shown as a stacked bar chart. The linear discriminant analysis (LDA) effect magnitude (LEfSe) method was used to detect differences in bacterial group abundance among the Ctrl, Hyp, and Rec groups. PICRUSt2 analysis was used to predict the function of intestinal flora by KEGG functional annotation.

### 2.9. Transcriptome and Intestinal Microorganism Correlation Analyses

To detect correlations between intestinal bacteria and the MAPK signaling pathway, Pearson’s correlation analysis was carried out using OmicStudio tool (https://www.omicstudio.cn (accessed on 15 August 2022)) [22]. The *p* value was corrected for multiple comparisons using R (version 3.6.1); *p* < 0.05 was considered to be statistically significant, *p* < 0.01 was very significant, and *p* < 0.001 was extremely significant.

### 2.10. Statistical Analysis

Data for 96h-LH_50_, serum biochemical parameters, and qRT-PCR were analyzed using SPSS 23.0 (SPSS Inc., Chicago, IL, USA) and are expressed as means ± standard error. Shapiro–Wilk and Levene median tests were used to test for variance normality and homogeneity of data. One-way analysis of variance (ANOVA) and Tukey’s test were used to detect significant differences in biochemical indices and gene transcript levels among different sampling times. The Kruskal–Wallis method was used to detect significant differences in alpha and beta diversity indices among different samples.

## 3. Results

### 3.1. Determination of 96h-LH_50_ in Largemouth Bass

**Figure 1:**
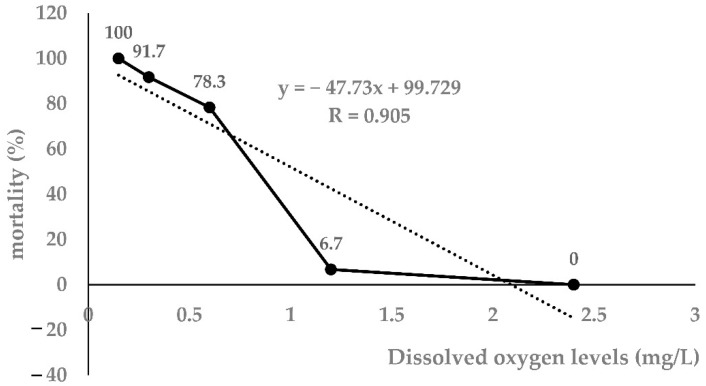
Linear interpolation graph for 96h-LH_50_ (*n* = 20).

As shown in Table 1, as the DO level decreased, the mortality of largemouth bass increased significantly. Taking the 96 h DO level and mortality as independent and dependent variables, respectively, the regression equation was obtained: Y = −47.73x + 99.72 (r = 0.905, *p* < 0.0001). The 96h-LH_50_ determined by linear interpolation was 1.04 mg/L (Figure 1). Therefore, 1.00 mg/L was selected as the 96h-LH_50_ of largemouth bass for further experiments.

### 3.2. Determination of Antioxidant Capacity of Largemouth Bass under Hypoxic Stress and Reoxygenation

**Figure 2:**
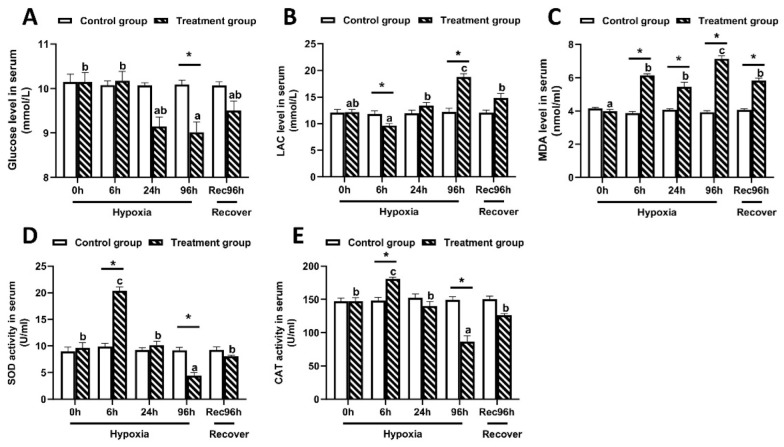
Serum biochemical indices of largemouth bass under 96 h of hypoxic stress and recovery for 96 h (*n* = 12). (**A**): Glucose concentration. (**B**): Lactic acid concentration. (**C**): Malondialdehyde (MDA) content. (**D**): Superoxide dismutase (SOD) activity. (**E**): Catalase (CAT) activity. Different letters above bars indicate significant differences among sampling times. Asterisks indicate significant difference between control group and hypoxia treated group at each time point.

As shown in Figure 2, the serum glucose content in largemouth bass reached the lowest level at 96 h under hypoxic stress, and then increased somewhat after 96 h of recovery under sufficient DO conditions (Figure 2A). In contrast, the LAC and MDA contents peaked at 96 h of hypoxic stress, and then decreased significantly by 96 h of recovery under sufficient DO conditions (Figure 2B,C). The activities of SOD and CAT reached the lowest values at 96 h of hypoxic stress, but significantly increased after 96 h of recovery under sufficient DO conditions (Figure 2D,E). All of these indices showed significant differences between the control group and the hypoxia group at 96 h (*p* < 0.05).

### 3.3. Intestinal Histological Structure of Largemouth Bass under Hypoxic Stress and Reoxygenation

**Figure 3:**
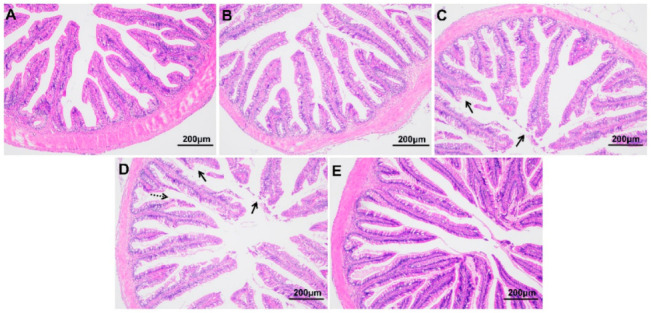
Intestinal morphology of largemouth bass under hypoxic stress for 0 h (**A**), 6 h (**B**), 24 h (**C**), 96 h (**D**) and 96 h of recovery under sufficient DO conditions (**E**) (*n* = 12). Solid arrow: intestinal epithelial injury; dotted arrow: ruptured intestinal villus.

As shown in Figure 3, compared with fish at 0 h, those exposed to hypoxic stress for 24 h and 96 h showed obvious tissue damage, including exfoliation of the chorionic epithelium, rupture of chorionic villi, and decreased thickness of the intestinal muscle layer (*p* < 0.05) (Table 2). The morphology of the intestine recovered somewhat after 96 h of recovery under sufficient DO conditions. The length and width of villi were not significantly affected by the hypoxia treatment in this experiment (*p* > 0.05).

### 3.4. Intestinal Cell Apoptosis in Largemouth Bass under Hypoxic Stress and Recovery

**Figure 4:**
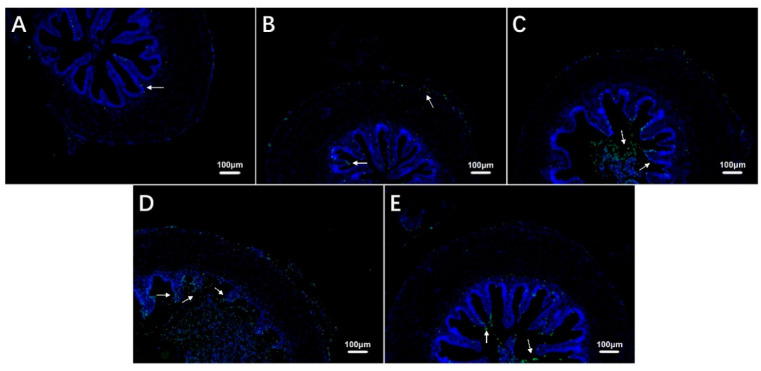
Effects of hypoxic stress (0 h (**A**), 6 h (**B**), 24 h (**C**), 96 h (**D**)) and reoxygenation (96 h (**E**)) on intestinal cell apoptosis in largemouth bass (*n* = 12). White arrows indicate apoptotic cells.

The TUNEL staining results showed that the number of apoptotic cells in the intestine of largemouth bass increased during exposure to hypoxia compared with the control group (Figure 4A–D, Table 3) and then decreased during recovery under sufficient DO conditions (Figure 4E, Table 3).

### 3.5. Transcriptome Analysis of Largemouth Bass under Hypoxic Stress and Reoxygenation

Nine libraries (Ctrl1, Ctrl2, Ctrl3, Hyp1, Hyp2, Hyp3, Rec1, Rec2, Rec3) were constructed from mRNA extracted from intestinal tissues of fish in the Ctrl (0 h), Hyp (96 h of hypoxic stress), and Rec (96 h recovery after hypoxic stress) groups. A total of 36,223,342–5,000,644 reads were obtained by sequencing. After quality-control steps to remove low-quality sequences, as shown in Table S2, the number of reads in each library ranged from 31,738,670 to 46,246,716 (Q20 value, 99.96–99.97%; Q30 value, 98.09–98.26%). The GC content was 47–49%, indicating that the sequencing data were of sufficient quality for subsequent analyses.

**Figure 5:**
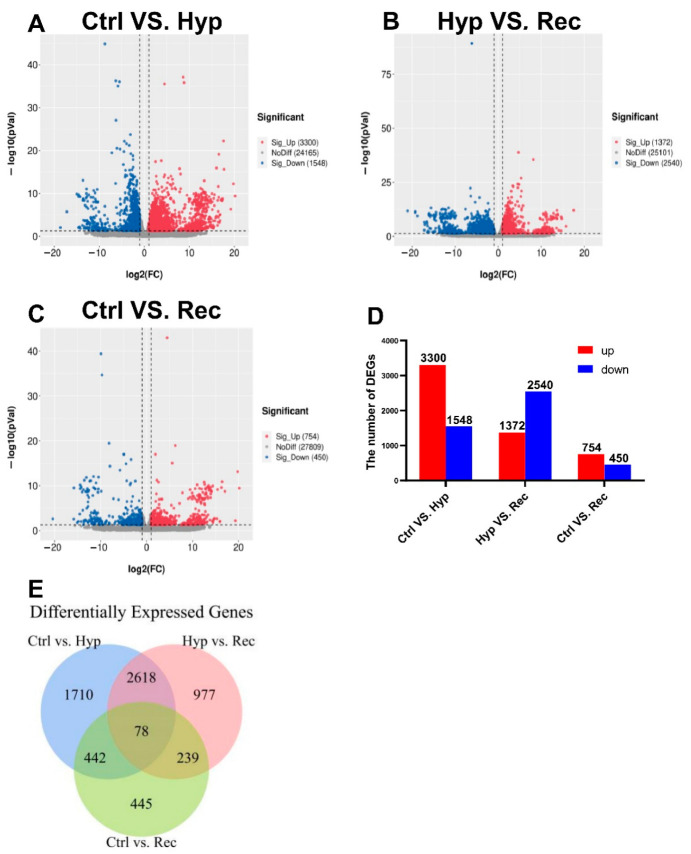
(**A**): Volcanic diagram of differentially expressed genes (DEGs) in Ctrl vs. Hyp groups (*n* = 3). (**B**): Volcanic map of DEGs in Hyp vs. Rec groups (*n* = 3). (**C**): Volcanic map of DEGs in Ctrl vs. Rec groups (*n* = 3). Grey dots represent genes with no significant difference in transcript levels between groups; red dots and blue dots represent significantly upregulated and downregulated DEGs, respectively. (**D**): Transcriptome analysis of DEGs quantity and expression (*n* = 3). (**E**): Venn diagram showing number of DEGs between and among groups (*n* = 3).

We detected 3300 upregulated DEGs and 1548 downregulated DEGs between the Ctrl group and the Hyp group (Figure 5); 1372 upregulated DEGs and 2540 downregulated DEGs between the Hyp group and the Rec group; and 1372 upregulated DEGs and 2540 downregulated DEGs between the Ctrl group and the Rec group (Figure 5A–D). The Venn diagram shows the number of DEGs between and among the different groups (Figure 5E).

The results of GO functional annotation analyses showed how hypoxic stress affected different subcategories of genes within the “biological process” (BP), “cellular component” (CC), and “molecular function” (MF) categories in largemouth bass (Figure S1). Compared with the Ctrl group, the Hyp group showed significant enrichment of DEGs in signal transduction, G-protein-coupled receptor signaling pathway, and oxidation-reduction process subcategories in the BP category; integral component of membrane, membrane, and cytoplasm subcategories in the CC category; and transferase activity, metal ion binding, and ATP-binding subcategories in the MF category. Compared with the Rec group, the Hyp group showed significant enrichment of DEGs in the regulation of transcription, DNA-template, and oxidation-reduction process subcategories in the BP category; membrane, integral component of membrane, and nucleus subcategories in the CC category; and metal ion binding, ATP binding, and transferase activity in the MF category. Compared with the Ctrl group, the Rec group showed significant enrichment of DEGs in the signal transduction, regulation of transcription, DNA-template, and proteolysis subcategories in the BP category; membrane, integral component of membrane, and cytoplasm subcategories in the CC category; and metal ion binding, ATP-binding, and nucleotide-binding subcategories in the MF category.

**Figure 6:**
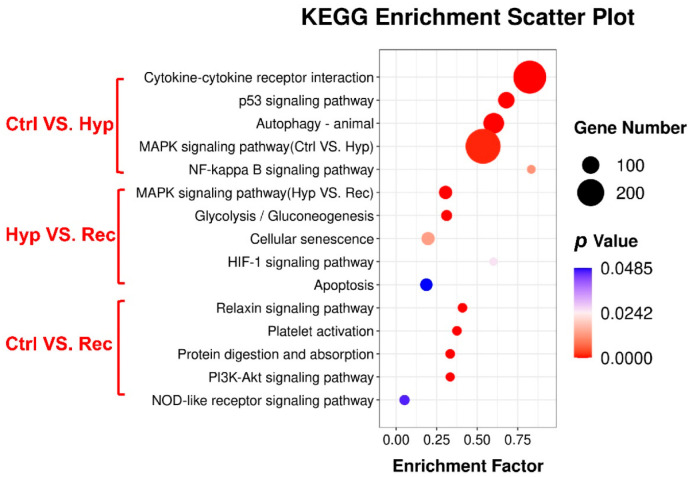
KEGG pathway enrichment analysis of DEGs in the intestine of largemouth bass under acute hypoxic stress (*n* = 3).

KEGG enrichment analysis showed that 114, 36, and 25 signaling pathways were enriched with DEGs in the Ctrl vs. Hyp, Hyp vs. Rec, and Ctrl vs. Rec comparisons, respectively. The oxidative stress pathways (HIF-1 signaling, MAPK signaling), inflammation pathways (MAPK signaling, NF-kappa B signaling, PI3K-Akt signaling), and apoptosis pathways (MAPK signaling, apoptosis, p 53 signaling) were significantly enriched with DEGs under hypoxia (*p* < 0.05) (Figure 6).

**Figure 7:**
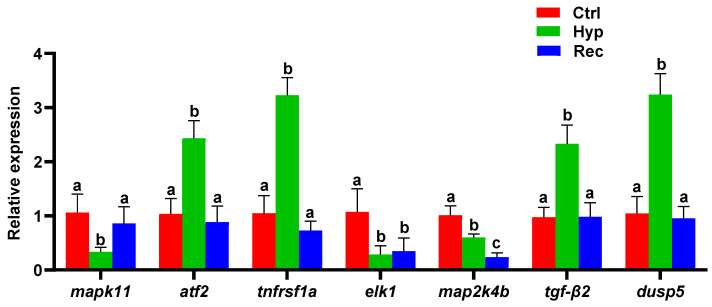
qRT-PCR analysis of differentially expressed genes in Ctrl group, Hyp group, and Rec group (*n* = 12). Different letters indicate significant differences in gene transcript levels among groups.

The MAPK signaling pathway was significantly enriched with DEGs in the Ctrl vs. Hyp and the Hyp vs. Rec comparisons. To verify these results concerning the immune and inflammatory responses of largemouth bass to hypoxic stress, several MAPK signaling pathway genes were selected for qRT-PCR analysis. The changes in gene transcript levels detected by qRT-PCR were consistent those detected from the sequencing results (Figure 7). Compared with the Ctrl group, the Hyp group showed significantly decreased transcript levels of *mapk11*, *elk-1,* and *map2k4b*. After 96 h of recovery under sufficient DO conditions, the transcript level of *mapk11* had increased, but the transcript level of *map2k4b* was still lower than that in the Hyp group. Compared with the Ctrl and Rec groups, the Hyp group showed significantly increased transcript levels of *atf2*, *tnfrsf1a*, *tgf-β2,* and *dusp5*.

### 3.6. Sequencing and Analysis of Intestinal 16S rRNA of Largemouth Bass under Hypoxic Stress and Reoxygenation

**Figure 8:**
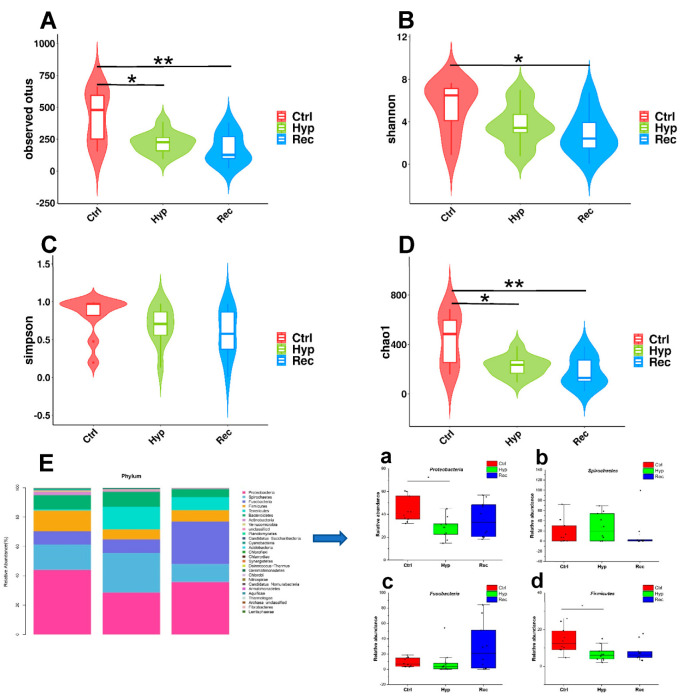
Intestinal alpha diversity of largemouth bass under hypoxic stress. (**A**): Observed operational taxonomic units (OTUs) index (alpha) (*n* = 10). (**B**): Shannon’s index (alpha) (*n* = 10), (**C**): Simpson’s index (alpha) (*n* = 10). (**D**): Chao1 index (alpha), * and ** indicate significant differences in abundance among different groups (Kruskal–Wallis test, *: *p* < 0.05, **: *p* < 0.01) (*n* = 10). (**E**): Relative abundance of main taxa of intestinal microflora in Ctrl group, Hyp group, and Rec group (**a**–**d**) (*n* = 10). Each bar represents relative abundance in each sample, and shows 27 most abundant taxa.

Alpha diversity is mainly used to reflect species richness and evenness as well as sequencing depth. Among the indices, Chao1 and observed OTUs mainly estimate the number of species in the community, while Shannon’s and Simpson’s indices reflect the diversity of the community. The results showed that the observed OTU index and Chao1 index were significantly lower in the Hyp group than in the Ctrl group (*p* < 0.05) (Figure 8A,D). The observed OTU index, Shannon’s index, and Chao1 index were significantly lower in the Rec group than in the Ctrl group (*p* < 0.05) (Figure 8A,B,D).

A PCoA analysis based on weighted UniFrac and unweighted UniFrac was used to describe the beta diversity of intestinal flora (Figure S2). The results indicated that there were significant differences in beta diversity among the Ctrl group, the Hyp group, and the Rec group (*p* < 0.05).

Proteobacteria, Spirochaetes, Fusobacteria, and Firmicutes were the dominant phyla in the intestinal microflora (Figure 8E). Under hypoxic stress, the abundance of Proteobacteria and Firmicutes decreased significantly in the Hyp group (*p* < 0.05) (Figure 8E(a,d)), while the abundance of Spirochaetes and Fusobacteria showed no significant differences among the three groups (Figure 8E(b,c)).

**Figure 9:**
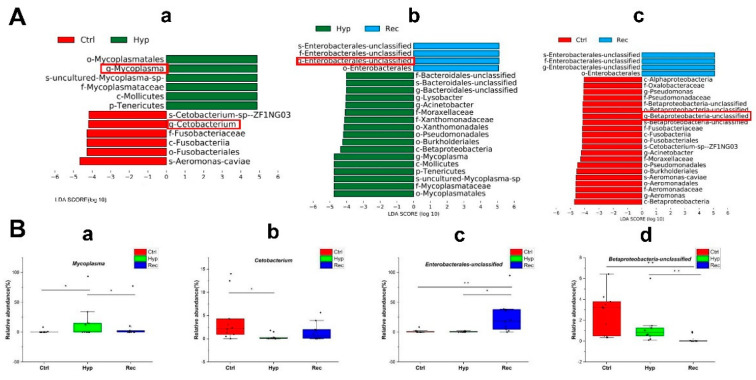
(**A**): Taxa shown in histogram were determined to differ significantly in abundance among Ctrl, Hyp, and Rec groups by Kruskal–Wallis test ((*p* < 0.05, LDA score > 4, (**a**): Ctrl vs. Hyp; (**b**): Hyp vs. Rec; (**c**): Ctrl vs. Rec) (*n* = 10). (**B**): Abundance of major bacteria (**a**–**d**) in Ctrl, Str, and Rec groups (*, *p* < 0.05; **, *p* < 0.01) (*n* = 10).

The LEfSe method was used to compare the abundance of all detected bacterial groups among the Ctrl, Hyp, and Rec groups (Figure S3). To determine which bacteria responded to differences in DO levels, we calculated the relative abundance of selected bacteria. The genera showing large differences in relative abundance among the three groups were Mycoplasma, Cetobacterium, unclassified Enterobacterales, and unclassified Betaproteobacteria. The relative abundance of Mycoplasma was significantly higher in the Hyp group than in the Ctrl and Rec groups (Figure 9B(a)). The relative abundance of Cetobacterium in the Hyp group was significantly lower than that in the Ctrl group (*p* < 0.05), but not significantly different from that in the Rec group (*p* > 0.05) (Figure 9B(b)). Enterobacteriaceae were more abundant in the Rec group than in the Ctrl and Hyp groups (Figure 9B(c)), while Betaproteobacteria were less abundant in the Rec group than in the Ctrl and Hyp groups (Figure 9B(d)).

**Figure 10:**
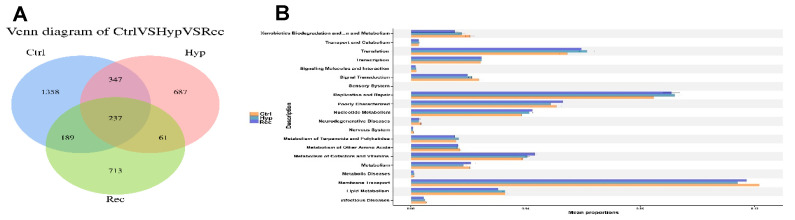
(**A**): Venn diagram showing number of OTUs shared between and among Ctrl, Hyp, and Rec groups (*n* = 10). (**B**): Abundance ratio of intestinal microflora in Ctrl, Hyp, and Rec groups with predicted level 2 functions (*n* = 10).

As shown in Figure 10A, the Ctrl, Hyp, and Rec groups shared 237 OTUs. Compared with the Ctrl group, the Hyp group had 748 unique OTUs and the Rec group had 774 unique OTUs. The main functions of the intestinal microorganisms at KEGG level 2 are shown in Figure 10B. The main functions of the intestinal microorganisms across the Ctrl, Hyp, and Rec groups were “membrane transport”, “replication and repair”, and “translation”.

### 3.7. Correlations between Intestinal Microorganisms and DEGS

**Figure 11:**
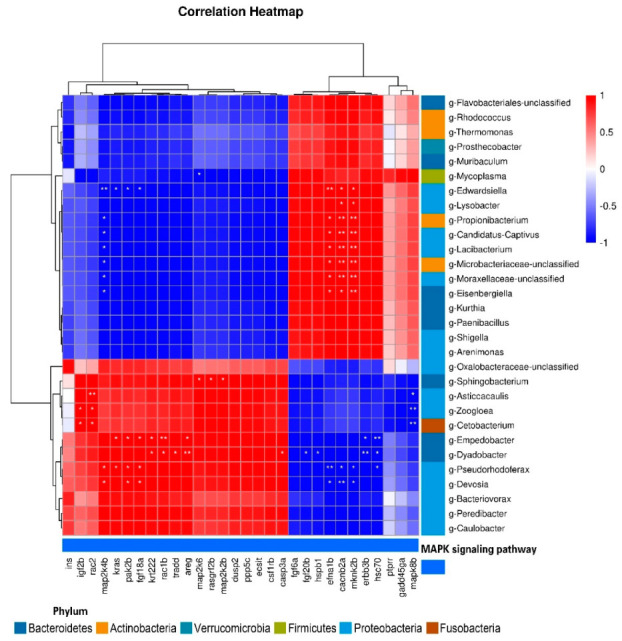
Correlation between DEGs and intestinal microflora at the genus level (*, *p* < 0.05; **, *p* < 0.01).

Pearson’s correlation analyses were conducted to detect relationships between DEGs and intestinal microorganisms at the genus level (Figure 11). We detected positive correlations between *igf2b* and *g-Zoogloea* and *g-Cetobacterium*; and between *rac2* and *g-Asticcacaulis*, *g-Zoogloea*, and *g-Cetobacterium*; and detected negative correlations between *mapk8b* and *g-Asticcacaulis*, *g-Zoogloea*, and *g-Cetobacterium*. *map2k4b* was positively correlated with *g-Devosia* and *g-Pseudorhodoferax* and negatively correlated with *g-Eisenbergiella*, *g-Moraxellaceae*, *g-Lacibacterium*, *g-Microbacteriaceae*, *g-Propionibacterium,* and *g-Edwardsiella*. The relationships between *efna1b*, *cacnb2a*, *mknk2b* and these flora were opposite to that of *map2k4b*. *cacnb2a* and *mknk2b* were positively correlated with *g-Lysobacter*. *kras* was positively correlated with *g-Pseudorhodoferax* and *g-Empedobacter*, and negatively correlated with *g-Edwardsiella*. *pak2b* and *fgf18a* were positively correlated with *g-Empedobacter*, *g-Devosia,* and *g-Pseudorhodoferax*. *krt222*, *areg,* and *rac1b* were significantly positively correlated with *g-Empedobacter* and *g-Dyadobacter*, while *hsc70* and *erbb3b* were significantly negatively correlated with *g-Empedobacter* and *g-Dyadobacter*. *hsc70* was negatively correlated with *g-Pseudorhodoferax*. *tradd* and *casp3a* were positively correlated with *g-Dyadobacter*, while *fgf20b* and *hspb1* were negatively correlated with *g-Dyadobacter*. *map2k6* was negatively correlated with *g-Mycoplasma*, and positively correlated with *g-Sphingobacterium. rasgrf2b* and *map2k2b* were also positively correlated with *g-Sphingobacterium.*

## 4. Discussion

The physiological state of fish is readily affected by water quality. The DO level is an important indicator of whether the water is suitable for fish survival or not. Hypoxic conditions affect the survival of fish and disrupt their normal physiological functions. Therefore, low DO is one of the main factors causing economic losses in the aquaculture industry [35]. In this study, the effects of hypoxic stress on the antioxidant system, immunity responses, and intestinal microflora of largemouth bass were studied through comprehensive analyses of serum, the intestine, and the intestinal microbiota.

Fish adapt to hypoxic stress via a series of complex physiological and biochemical changes [1]. The concentrations of glucose and lactic acid in serum are readily affected by external pressure. Hypoxia leads to stress in fish: there is a change from aerobic metabolism to anaerobic metabolism, and anaerobic glycolysis increases, resulting in the accumulation of lactic acid in serum [36,37]. In the present study, we found that blood glucose levels were lower in the hypoxia-treated group than in the control group. It may be that fish consume more endogenous substances such as glucose during the response to hypoxic stress, leading to a lack of energy. When fish lack energy, their ability to resist stress may be diminished. Many studies have shown that reactive oxygen species (ROS) are overproduced in fish under hypoxic conditions. The superoxide anion radical can be decomposed into H_2_O_2_, which is less harmful, via the activity of SOD, and then CAT decomposes H_2_O_2_ into H_2_O and O_2_ through the cellular antioxidant pathway [38]. Therefore, the activities of CAT and SOD reflect the defense function of the antioxidant system to some extent. Lipid peroxidation is one of the main forms of damage caused by oxidative stress, and the extent of lipid peroxidation is reflected by the content of MDA, the final product of this reaction. High levels of MDA can lead to further lipid peroxidation in the cell membranes and cell damage [39]. Studies on Nile tilapia (*Oreochromis niloticus*) and Chinese sea bass (*Lateolabrax maculatus*) subjected to hypoxic conditions for 24 h detected signs of oxidative stress in their intestines, gills, and liver, including increased activities of SOD and CAT [40]. After exposure to hypoxic conditions for 48 h, the activities of SOD and CAT decreased and the content of MDA increased [14]. Recovery under sufficient DO conditions restored the activity of SOD and CAT. Our findings suggest that hypoxic stress can lead to oxidative stress. More specifically, short-term hypoxia activates the antioxidant system, while long-term hypoxia leads to metabolic dysfunction and a decreased antioxidant capacity.

Previous studies have shown that oxidative stress can lead to excessive ROS production, damage to intestinal structure [41], cell apoptosis [42], and impaired function of the intestinal barrier [43]. Intestinal villus morphology and muscle thickness are important indices to measure the function of the intestinal barrier [13]. Previous studies have shown that hypoxia can significantly affect the morphology and thickness of the intestinal muscle layer [12,44]. The decreased muscle thickness observed in our study may indicate that hypoxia led to a loosening of the dense connective tissue of the muscle. We observed that the intestinal villous epithelium sloughed off, the villi broke, and the thickness of muscle layer decreased in fish under hypoxic stress. All of these results indicate that the function of the intestinal barrier in largemouth bass was impaired under hypoxic stress. Such extensive tissue damage may lead to intestinal inflammation and the invasion of opportunistic bacteria. Studies have shown that excessive accumulation of H_2_O_2_ under oxidative stress can induce cell apoptosis and lead to tissue damage [45]. In our study, the number of apoptotic intestinal cells had increased by 96 h of hypoxic stress, and this may have been related to the decrease in CAT activity under prolonged hypoxic conditions.

To explore the mechanisms underlying these biochemical and physiological changes in largemouth bass, we carried out transcriptome analyses. High-throughput sequencing revealed which regulatory pathways and key genes involved in those pathways were affected under hypoxic stress [46]. The response of cells to oxidative stress is guided by signal transduction pathways. The cascade of MAPK family members plays an important role in regulating cell survival and the immune response [47]. The transcriptome analysis showed that the MAPK signaling pathway was significantly enriched with DEGs under hypoxic stress. The MAPK signaling pathway is sensitive to extracellular stimulation. *mapk11* encodes p38β, which is one of the four members of the p38 subfamily, while *tgf-β2* and *map2k4b* are upstream and downstream genes of the MAPK pathway, respectively. Under external stimulation, both *tgf-β2* and *map2k4b* can rapidly activate JNK/p38 [48,49]. The targets of JNK/p38 include Ets-like transcription factor-1 (*elk-1*) and activating transcription factor 2 (*atf2*), and so the transcript levels of these genes change in response to the phosphorylation of JNK/p38 during the stress response. The product of *elk-1* inhibits apoptosis in the cytoplasm [50], while upregulation of *atf2* regulates the immune response [51] and protects the intestine from excessive damage caused by the destructive stimulus [52]. In this study, the downregulation of *elk-1* and the upregulation of *atf2* may be indicative of a self-protective response in fish to activate the immune system under hypoxic stress. The tumor necrosis factor receptor superfamily (*tnfrsf1*) can be divided into type I (*tnfrsf1a*) and type II (*tnfrsf1b*) family members. The product of *tnfrsf1a* mainly triggers apoptosis or inflammation [53]. Under hypoxic conditions, the overexpression of *tnfrsf1a* can promote the activation of the NF-κB signal pathway and induce inflammation [54]. Another study showed that during tissue inflammation, *dusp5* can specifically inhibit the phosphorylation of ERK1/2, thereby downregulating the expression of the proinflammatory gene *tnf-α*, whose expression pattern is parallel with that of *dusp5* [55]. Our results show that there is a similar phenomenon between *dusp5* and *tnfrsf1a* under hypoxic stress, suggesting that *dusp5* may reduce inflammation and cell apoptosis by simultaneously affecting *tnf-α* and its receptors.

Under stress conditions, fish intestinal flora can be used as a biomarker of the stress response [56]. The intestinal microflora inhabit the gastrointestinal tract and play an important role in maintaining the function of the intestinal mucosal barrier. The microflora can regulate homeostasis of the internal environment of fish, especially that of the intestinal tract, and are closely related to the antioxidant response, immune response, and pathogenic reactions of fish [57,58]. Under oxidative stress, mitochondrial and bacterial DNA are inserted into the nuclear genome, resulting in changes in cellular gene expression [59]. Previous studies have shown that hypoxic stress significantly affects fish gut microflora [60] and that gut microflora is associated with hypoxia tolerance [61]. In this study, we found that hypoxic stress affected the number of species, species richness, community composition in the intestinal microflora of largemouth bass [18,62], and this may have been related to the high level of ROS in the intestine of fish under oxidative stress [63]. A high ROS level is indicative of intestinal inflammation [64]. After 96 h of recovery under sufficient DO conditions, the number of anaerobes was greatly decreased, and the specific species of bacteria differed significantly from those in the Hyp group.

In this study, Proteobacteria, Spirochaetes, Fusobacteria, and Firmicutes were the dominant intestinal bacteria in the three groups. These results, combined with the results of the alpha and beta diversity analyses, show that hypoxia changed the structure of intestinal microflora communities in largemouth bass, but did not change the dominant species. Proteobacteria and Firmicutes showed significant changes in abundance under hypoxic stress.

Proteobacteria are Gram-negative bacteria that are very sensitive to environmental factors. Previous studies have shown that the expansion of Proteobacteria under stress is the main feature of intestinal inflammation [65]. However, in zebrafish (*Danio rerio*), hypoxia led to a significant decrease in the abundance of Proteobacteria [66], and this was related to inflammation [67]. We speculate that the subordinate flora of Proteobacteria are more obviously affected by oxygen concentration. In short, the imbalance of Proteobacteria in the intestinal tract sends a danger signal. Firmicutes is the main taxon in the intestinal flora of most vertebrates [68]. Members of this family are spherical or rod-shaped, with cell walls, and many of them are beneficial bacteria. Some studies have shown that Firmicutes contribute to the absorption of fatty acids in the intestine [69] and energy metabolism [70]. To some extent, a decrease in the abundance of Firmicutes reflects the imbalance of intestinal metabolism under hypoxic stress.

*Mycoplasma* and *Enterobacterales* are common pathogens in humans and animals [71], and they can strongly induce an inflammatory response in the intestine [72,73]. The increased abundance of these taxa in the Hyp and Rec groups suggests an increased risk of intestinal disease in largemouth bass under hypoxic stress, although there is usually a lag time before such disease outbreaks occur under stress conditions [22]. *Cetobacterium* is classified as a Fusobacterium. This bacterium was found to improve glucose homeostasis through parasympathetic activation in zebrafish [74]. As a probiotic, *Cetobacterium* was found to improve intestinal health and enhance resistance to pathogens in tilapia [75]. Previous studies have also shown that the abundance of *Cetobacterium* is significantly affected under hypoxic stress [66]. Overall, our results show that the abundance of beneficial intestinal bacteria decreases and the abundance of opportunistic pathogens increases under hypoxic stress. Thus, hypoxic stress may cause intestinal inflammation via disruption of the intestinal flora.

We detected differences in microbial community structure among the Ctrl, Hyp, and Rec groups, indicating that the host genome interacted with the microbiome to select for certain microbial taxa [20]. The combined results of intestinal microflora and transcriptome analyses revealed relationships between some microorganisms and DEGs in the MAPK signaling pathway. *Edwardsiella* is an opportunistic pathogen in the gut. A significant increase in the abundance of *Edwardsiella* was detected in the intestine of zebrafish with enteritis [76]. In this study, *Edwardsiella* was significantly associated with several genes with different functions, which indicates the complexity of the response of largemouth bass to hypoxic stress. We also detected a significant correlation between Proteobacteria and MAPK pathway genes, indicating that intestinal flora respond to acute host immune activation by rapidly changing gene transcription when the host undergoes pathological changes [77]. In conclusion, our results show that the intestinal inflammatory response of largemouth bass under hypoxic stress may be mediated by the MAPK signaling pathway, and this may be related to the disruption of the intestinal microflora.

## 5. Conclusions

Hypoxic stress caused oxidative stress, decreased antioxidant capacity, induced inflammatory and apoptotic responses in the intestine, activated the MAPK signaling pathway, and disrupted the intestinal microflora of largemouth bass. Ultimately, these changes resulted in impaired function of the intestinal barrier. The results of this study provide new insights into how fish adapt to hypoxic stress.

## Figures and Tables

**Table 1:** Effects of different low DO levels on 96 h mortality (%) of largemouth bass.

Time (h)	Dissolved Oxygen Levels (mg/L)				
	2.4	1.2	0.6	0.3	0.15
0	0	0	0	0	0
24	0	0	20	31.7	50
48	0	0	46.7	58.3	73.3
72	0	0	68.3	78.3	100
96	0	6.7	78.3	91.7	100

**Table 2:** Morphological indices of largemouth bass under hypoxic stress followed by 96 h of recovery.

	Hyp_0 h	Hyp_6 h	Hyp_24 h	Hyp_96 h	Rec_96 h
Villi Length (μm)	654.85 ± 54.48	630.65 ± 42.79	452.70 ± 52.72	498.94 ± 61.55	518.87 ± 94.61
Villi Thickness (μm)	93.37 ± 6.48	89.14 ± 7.11	75.05 ± 2.36	76.77 ± 1.45	78.80 ± 5.6
Muscle Layer Thickness (μm)	83.34 ± 3.37 ^a^	67.09 ± 7.46 ^ab^	53.87 ± 4.90 ^bc^	42.01 ± 3.91 ^c^	64.04 ± 4.62 ^abc^

^a, b, c^ Within each row, different letters indicate significant differences among groups.

**Table 3:** Number of intestinal apoptotic cells of largemouth bass under hypoxic stress followed by 96 h of recovery.

	Hyp_0 h	Hyp_6 h	Hyp_24 h	Hyp_96 h	Rec_96 h
Number of Apoptotic Cells	18.42 ± 4.09 ^a^	75.33 ± 12.45 ^a^	291.58 ± 35.16 ^b^	978.08 ± 237.68 ^c^	333.08 ± 31.99 ^b^

^a, b, c^ Within each row, different letters indicate significant differences among groups.

## Data Availability

The data are contained within the article and Supplementary Materials.

## Supplementary Materials

The following supporting information can be downloaded at: https://www.mdpi.com/article/10.3390/antiox12010001/s1, Table S1. Sequences of primers used for qRT-PCR; Table S2. Overview of transcriptome sequencing reads and quality filtering of largemouth bass; Figure S1. GO classification of Ctrl, Hyp and Rec DEGs; Figure S2. Btea diversity was analyzed based on weighted UniFrac(A) and unweighted UniFrac(B) PCOA analysis; Figure S3. Taxonomic cladistics derived from Lefse analysis. Red, green, and blue represent Ctrl, Hyp, and Rec groups, respectively. The brightness is proportional to the abundance of taxa.
