# Supplementary material for: Transcriptome and 16S rRNA Analyses Reveal That Hypoxic Stress Affects the Antioxidant Capacity of Largemouth Bass (Micropterus salmoides), Resulting in Intestinal Tissue Damage and Structural Changes in Microflora
Source: Antioxidants (Basel). 2022 Dec 20;12(1):1. doi: 10.3390/antiox12010001 (PMC9854696; doi:10.3390/antiox12010001)
Supplement: Supplementary file 1 [file antioxidants-12-00001-s001.zip › antioxidants-2075942-supplementary.pdf]

Supplementary Materials:

Table S1. Sequences of primers used for qRT-PCR.

Name	Accession number	Primer sequence (5′-3′)
mapk11	XM_038738930.1	F: TTACGACAGAAGGTTGCGGT
		R: CTAATGTTGCGGCAGGTGTG
atf2	XM_038720530.1	F: TACGATGGGCTTGTGTTCCG
		R: GAGTCACGGAAAGAGTGCGA
tnfrsf1a	XM_038711147.1	F: CTTGGCAGAGAGGGGCAAC
		R: CATCTGAGGTCAACTGGACGG
elk1	XM_038721756.1	F: ATCCACGGCTCGGACATTTT
		R: AACAGACGACGAGCGAAGAG
map2k4b	XM_038735280.1	F: GCAGCAGCATGCAAGGTAAA
		R: CGCTCTATGTGAGGGTTCTGG
tgf-β2	XM_038710299.1	F: GCACGGTTTGCAGGAATTGA
		R: GACTCCAGCCGGTATGAAGG
dusp5	XM_038713959.1	F: GATGCGGCCTTCGACATCAT
		R: GCGTGGTAGGTTCAGGTGTG
β-actin	XM_038695351.1	F: ATCGCCGCACTGGTTGTTGAC
		R: CCTGTTGGCTTTGGGGTTC

Table S2. Effects of different low dissolved oxygen levels on 96 h mortality (%) of largemouth bass. overview of tran scriptome sequencing reads and quality filtering of largemouth bass.

Sample	Raw Data		Valid Data		Valid Ratio (reads)	Q20%	Q30%	GC content%
	Read	Base	Read	Base				
Ctrl1	50006644	7.50G	46246716	6.94G	92.48	99.96	98.17	47.50
Ctrl2	42367108	6.36G	38108322	5.72G	89.95	99.97	98.19	49
Ctrl3	39878200	5.98G	36515778	5.48G	91.57	99.96	98.13	48.50
Hyp1	44148112	6.62G	39657008	5.95G	89.83	99.97	98.16	48
Hyp2	45814852	6.87G	41195552	6.18G	89.92	99.97	98.09	48
Hyp3	42749920	6.41G	36835290	5.53G	86.16	99.97	98.13	48
Rec1	36223342	5.43G	31738670	4.76G	87.62	99.96	98.18	47.50
Rec2	43310240	6.50G	40021578	6.00G	92.41	99.96	98.15	47
Rec3	46460538	6.97G	42624682	6.39G	91.74	99.97	98.26	47.50

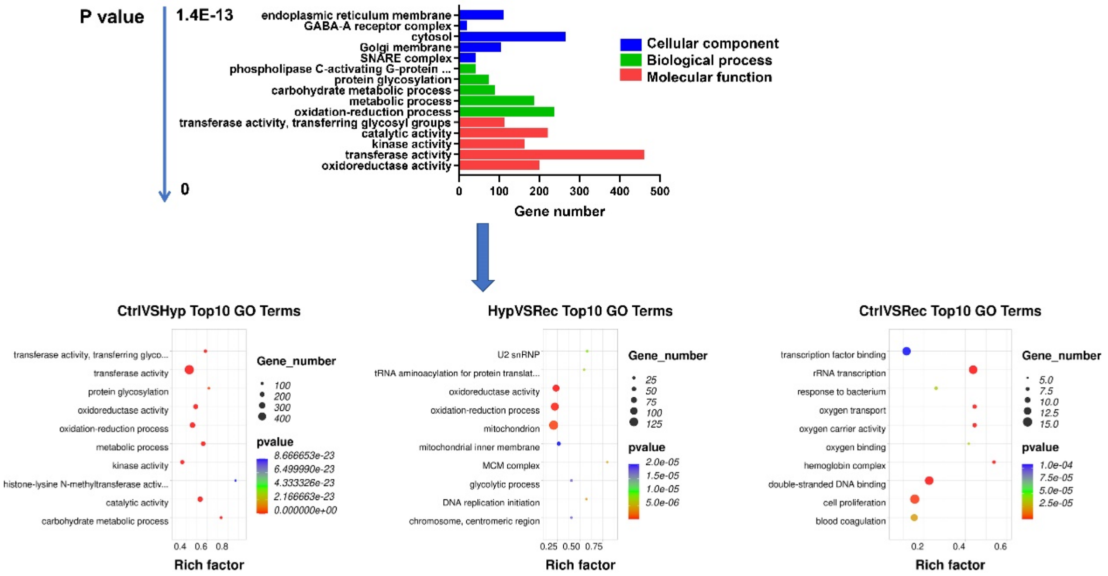

Figure S1. GO classification of Ctrl, Hyp and Rec DEGs.

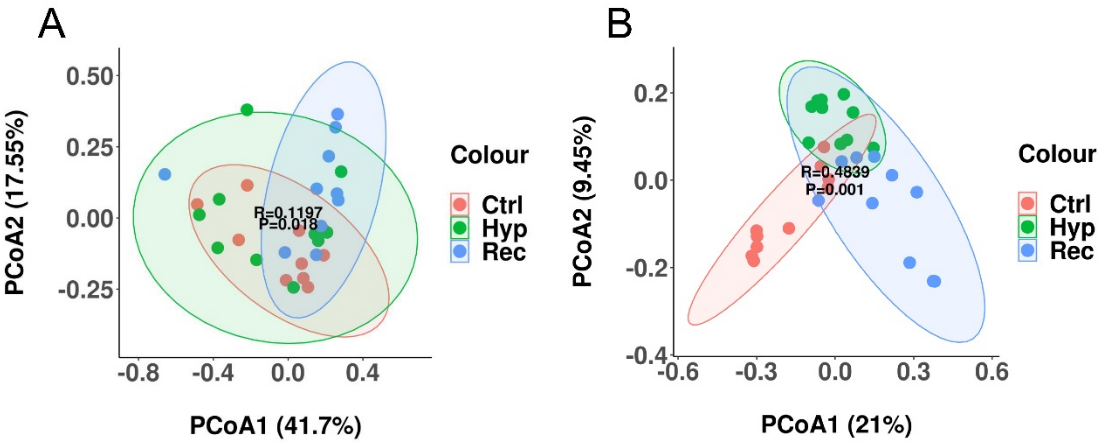

Figure S2. Btea diversity was analyzed based on weighted UniFrac(A) and unweighted UniFrac(B) PCOA analysis.

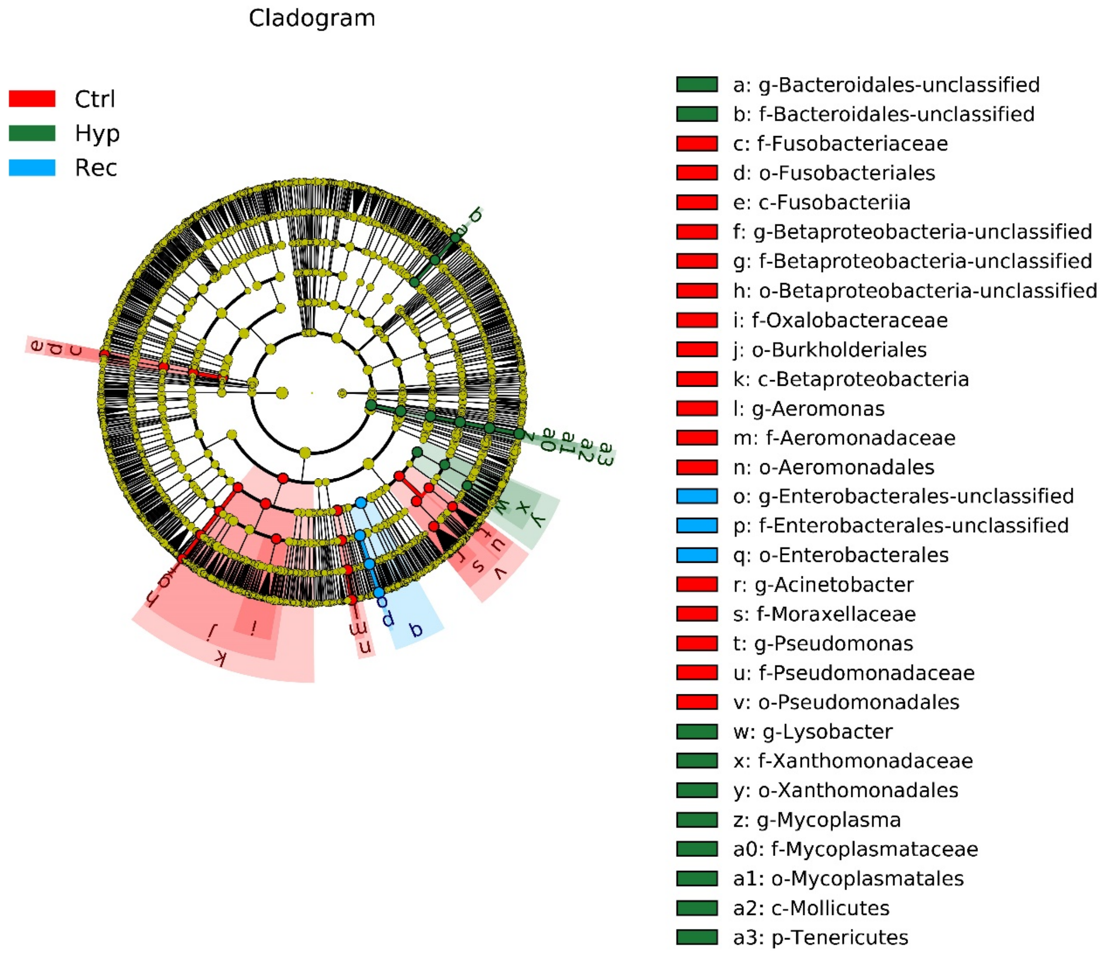

Figure S3. Taxonomic cladistics derived from Lefse analysis. Red, green, and blue represent Ctrl, Hyp, and Rec groups, respectively. The brightness is proportional to the abundance of taxa.
